# Laparoscopic ovariectomy provides favourable peri‐ and postoperative outcomes compared with ovariohysterectomy via celiotomy in bitches

**DOI:** 10.1111/jsap.70013

**Published:** 2025-08-20

**Authors:** R. Moxon, E. Yarwood, H. Hawkins, J. Came, G. C. W. England

**Affiliations:** ^1^ Canine Science, Guide Dogs National Centre Leamington Spa UK; ^2^ School of Veterinary Medicine and Science University of Nottingham Sutton Bonington UK; ^3^ Feldon Veterinary Centre Leamington Spa UK

## Abstract

**Objectives:**

To investigate the peri‐ and postoperative outcomes for bitches neutered via laparoscopic ovariectomy or open ovariohysterectomy (celiotomy).

**Materials and Methods:**

Retrospective data were obtained for 519 bitches neutered in UK Veterinary Practices (VP) by either laparoscopic ovariectomy (LAP‐OVE, *n* = 213, performed at VP1) or ovariohysterectomy by celiotomy (OVH‐CEL, *n* = 306, performed at VP1‐4). Data for complications in the perioperative and postoperative periods were compared for LAP‐OVE, all OVH‐CEL, and VP1 OVH‐CEL (providing a comparison between procedures at the same practice). Differences in surgery duration were examined using generalised linear mixed models.

**Results:**

There was no significant difference in surgery duration between LAP‐OVE surgeries and all OVH‐CEL surgeries, or VP1 OVH‐CEL surgeries. Intraoperative complications were reported for 2.3% of LAP‐OVE, 2.9% of all OVH‐CEL and 1.7% of VP1 OVH‐CEL surgeries. Postoperative complications prior to being discharged were reported for 0.5% of LAP‐OVE, 1.0% of all OVH‐CEL and 0% of VP1 OVH‐CEL surgeries. There were fewer LAP‐OVE than OVH‐CEL bitches with redness or swelling of the wound site in the postoperative period (16.3% LAP‐OVE, 36.6% all OVH‐CEL and 43.1% of VP1 OVH‐CEL surgeries). Complications in the postoperative period that required veterinary attention were reported for 12.9% of LAP‐OVE, 16.3% of all OVH‐CEL and 24.0% of VP1 OVH‐CEL bitches. Inappetence, discomfort and uncharacteristic licking or chewing of parts of the body were observed in fewer bitches following laparoscopic ovariectomy than following ovariohysterectomy by celiotomy.

**Clinical Significance:**

The findings provide evidence to support the contention that postoperative complications, including those related to wound healing and those requiring further veterinary intervention, are lower following laparoscopic than open neutering surgeries in bitches.

## INTRODUCTION

Ovariohysterectomy (OVH) and ovariectomy (OVE) are accepted methods of neutering bitches in the UK, performed by either celiotomy or increasingly laparoscopically (Romagnoli et al., 2024; Rosewell, 2016; Woodlands, 2018). Deciding whether and through which method to neuter bitches depends greatly on veterinary opinions on the beneficial and detrimental impacts on peri‐surgical complications and immediate postoperative recovery, as well as the long‐term health and behavioural outcomes (Hoad, 2018; Howe, 2015; Yates & Leedham, 2019).

Peri‐ and postoperative complications associated with surgical neutering can include stress, pain, short‐term anaesthetic risks, unusual postoperative behaviour and problems with wound healing and discomfort (Moxon et al., 2023; Väisänen, 2006). Haemorrhage has been reported as the most common complication observed during OVH (Belluzzi et al., 2022; Berzon, 1979; Burrow et al., 2005; Muraro & White, 2014; Romagnoli et al., 2024). Complications reported less frequently include problems with anaesthesia, accidental ureteral injury or ligation and trauma to the intestines or spleen (Airikkala‐Otter et al., 2018; Belluzzi et al., 2022; Benavides Melo et al., 2018; Muraro & White, 2014). Intraoperative dropping of ovaries in extraction, haemorrhaging, tissue trauma in port placement, gas embolism and splenic puncture are identified as complications associated with laparoscopic neutering surgeries (Nylund et al., 2017; Tapia‐Araya et al., 2015; Wormser & Runge, 2016).

The surgical wound is the most frequently reported site of postoperative complications, and wound healing is associated with complications more often than any perioperative problem (Adin, 2011; Burrow et al., 2005; Muraro & White, 2014). Wound healing complications present as swelling, discharge, seroma, granulomatous reaction and self‐inflicted trauma (Akinrinmade & Eyarefe, 2012; Berzon, 1979; Blacklock et al., 2016; Burrow et al., 2005; Howe, 1997; Muraro & White, 2014; Pollari & Bonnett, 2006). Peri‐ and postoperative complications are suggested to be influenced by surgeon experience and technique (Blacklock et al., 2016; Howe, 2006; Peeters & Kirpensteijn, 2011), by dog breed, with congenital breed impediments increasing the risk of intra‐surgical complications (Kennedy & Smith, 2014), and by length of surgery (Airikkala‐Otter et al., 2018; Beal et al., 2000; Burrow et al., 2005; Nicholson et al., 2002).

Some studies have compared OVE and OVH surgeries. Significantly greater skin incision lengths were reported for open OVH than for OVE neutering surgeries in two randomised clinical trials; although in both studies, there was no difference in postoperative pain scores (Peeters & Kirpensteijn, 2011; Tallant et al., 2016). Significantly shorter surgery durations have been reported for open OVE (*n* = 10) than for OVH (*n* = 10; Tallant et al., 2016) and for laparoscopic OVE (*n* = 147) and OVH (*n* = 131) neutering surgeries (Corriveau et al., 2017); although in both of these studies, there was no controlling for confounding variables in the analysis. Peeters and Kirpensteijn (2011) found no difference in surgery duration between open OVE (*n* = 20) and OVH (*n* = 20) surgeries when bodyweight, body condition score and sternal manubrium‐to‐pubic rim distance were included in the analysis. No significant differences were identified in any of the three studies relating to peri‐ or postoperative complications or pain scores.

For studies that have compared laparoscopic and more traditional techniques, such as celiotomy, findings related to surgery duration varied. Davidson et al. (2004) reported significantly longer surgery durations for laparoscopic (*n* = 16) versus open OVH (*n* = 18) surgeries, and Culp et al. (2009) reported a similar finding for laparoscopic (*n* = 10) versus open OVE (*n* = 10) surgeries. However, Shariati et al. (2014) reported significantly shorter surgery durations for laparoscopic (*n* = 8) than for open (*n* = 8) OVE surgeries. None of these clinical trials controlled for confounding variables in their analyses, and surgeon experience was not clearly stated. Devitt et al. (2005) reported no difference in surgical time between bitches of similar ages and weights neutered by laparoscopic (*n* = 10) versus open OVH (*n* = 10). In the only retrospective cohort study identified that examined the differences between laparoscopic (*n* = 154) and open OVE (*n* = 106) surgeries, surgery duration was not considered. Lower overall and wound‐related complication rates were reported for the laparoscopic (20% overall and 11% wound healing) than the open surgical group (44% overall and 28% wound healing; Charlesworth & Sanchez, 2019). However, this study was not randomised, and no statistical analyses were conducted. In other studies, low numbers of peri‐ and postoperative complications have been reported, with no significant differences between laparoscopic and open surgeries (Culp et al., 2009; Davidson et al., 2004; Devitt et al., 2005; Shariati et al., 2014), although greater blood loss during surgery and higher pain scores have been reported for open than for laparoscopic surgeries (Davidson et al., 2004; Devitt et al., 2005; Shariati et al., 2014).

This study aimed to investigate the peri‐ and postoperative outcomes in bitches neutered via laparoscopic ovariectomy or open ovariohysterectomy (celiotomy), anecdotally reported as the two methods of neutering bitches performed most frequently in the UK.

## MATERIALS AND METHODS

### Study design

A retrospective cohort study was undertaken using existing data for 519 bitches neutered by either laparoscopic ovariectomy (LAP‐OVE) or ovariohysterectomy by celiotomy (OVH‐CEL). For the LAP‐OVE group, data were gathered from veterinary records for the surgical and post‐surgical periods. Data for the OVH‐CEL group were gathered as previously described (Moxon et al., 2023).

### Study setting

Two groups of bitches were identified that had been neutered using one of two surgical methods: bitches from the UK pet dog population that were neutered by laparoscopic ovariectomy and bitches from a UK assistance dog programme that had been neutered by open ovariohysterectomy (celiotomy). Surgeries were performed at one of four UK veterinary practices (VP). LAP‐OVE surgeries were performed at one veterinary practice (VP1) by experienced veterinary surgeons using the following protocol. Bitches were premedicated using acepromazine or medetomidine combined with suitable opiate analgesia such as methadone. An injection of non‐steroidal anti‐inflammatory was administered perioperatively. General anaesthesia was induced with propofol intravenously and, following intubation, was maintained with isoflurane in oxygen. A two‐port technique was used. The primary port was placed 1 to 2 cm caudal to the umbilicus in the midline using the modified Hasson technique. The abdomen was insufflated with medical carbon dioxide gas to a maximum pressure of 10 mmHg. A 5‐mm 0° rigid endoscope was used to examine the abdomen. The secondary port was placed 2 to 3 cm cranial to the umbilicus in the midline under direct visualisation. The bitch was rotated from dorsal recumbency to assist in visualising the ovaries. The ovaries were suspended using a Tankersley ovariectomy hook with a weighted handle. Following suspension, the ovarian pedicle was cauterised and transected using an advanced bipolar vessel sealing device (Ethicon ENSEAL). The stump was checked for bleeding, the bitch was returned to dorsal recumbency, and the ovaries were then removed via the secondary port site. The abdomen was deflated. The external rectus sheath at each port site was closed with synthetic absorbable suture (PDS). The skin was closed using either skin sutures of permanent non‐absorbable material (nylon) or intradermal sutures using suitable synthetic absorbable suture. All laparoscopic procedures were recorded to include the length of the procedure, maximum insufflation pressure and volume of carbon dioxide used.

All four VP (VP1‐4) performed OVH‐CEL surgeries following a standardised neutering protocol as previously described by Moxon et al. (2023). Briefly, bitches were premedicated using acepromazine or medetomidine combined with suitable opiate analgesia such as methadone and induced with propofol and maintained with isoflurane. Surgery involved a midline laparotomy with ligation of the ovarian vessels using synthetic absorbable suture material or Chromic Catgut, ligation (with the same material above) of the broad ligament and section with scissors, ligation (with the same material above) of the uterine vessels at the proximal cervix either individually followed by a circumferential ligation of the uterus or using a transfixion ligature that also ligates the uterus and transection of the caudal uterus. The uterine stump was left open and was returned to the abdominal cavity. The linea alba was closed using continuous or interrupted sutures, and the sub‐cutaneous fat layer was closed with a continuous suture pattern, in both cases using a synthetic absorbable suture. The skin was closed with simple interrupted or continuous sutures of a monofilament or a coated braided permanent material, or a continuous sub‐cuticular suture pattern using a synthetic absorbable sterile surgical suture.

Clinical information for the perioperative periods for LAP‐OVE bitches was extracted from data collected and recorded in Excel by VP1 to monitor laparoscopic procedures. Clinical information for the postoperative period for LAP‐OVE bitches was collected by examining the practice clinical records. For OVH‐CEL bitches, data from purpose‐designed data capture forms completed by the veterinarian at the time of surgery and by the bitches’ caregivers for the days following surgery were collected as part of a previous study (Moxon et al., 2023). Data included the date of surgery, the veterinarian performing the surgery, the complications reported during the surgery, in the immediate postoperative period and in the days following surgery (postoperative complications), and drug use (analgesia, anti‐inflammatory drugs and medication given to take home).

### Study participants

Data for bitches from an assistance dog programme as well as bitches of similar breeds and sizes from the pet dog population were used in the study.

Five hundred and nineteen bitches from six breeds and cross‐breeds were allocated to one of two surgery groups: LAP‐OVE (*n* = 213) and OVH‐CEL (*n* = 306). Breeds were reviewed and grouped for analysis. Any breed with fewer than 10 individual animals was grouped as “Other” (Table 1).

**Table 1:** The number of bitches of each breed in the study investigating the outcomes of neutering via two different surgical techniques [laparoscopic ovariectomy (LAP‐OVE) and ovariohysterectomy via celiotomy (OVH‐CEL)]

Breed	LAP‐OVE	OVH‐CEL
Golden retriever	23	0
Golden retriever cross labrador	2	306
German shepherd dog and German shepherd dog cross	23	0
Labrador	75	0
Other	71	0
Poodle cross	19	0
Total	213	306

### Variables and data sources

#### Perioperative outcomes

Information gathered in the immediate perioperative period included bitch bodyweight (kg), surgery duration (minutes), agreed with the four lead vets as “skin to skin,” as well as any perioperative complications and complications during anaesthesia, during recovery from anaesthesia and in the immediate postoperative period. Perioperative complications were not defined and were described by the veterinary surgeon in a free‐text box in the Excel sheet (LAP‐OVE) or data collection form (OVH‐CEL). Use of other anaesthetic protocols, analgesia and any other medications were recorded. Each bitch was awarded a perioperative complications score (out of four) that totalled the number of complications reported by veterinarians.

Perioperative outcome variables examined were (1) surgery duration (minutes), (2) intraoperative complications, (3) complications during anaesthesia, (4) complications during recovery from anaesthesia, (5) complications in the immediate postoperative period and (6) perioperative complications score. Confounding variables for analysis of surgery duration were breed and intraoperative complications as fixed factors; VP and veterinarian performing the surgery as random effects; and bodyweight and age at surgery as covariates.

#### Postoperative outcomes

Data for the postoperative period included the following: whether there was redness and swelling or discharge from the wound site; whether any veterinary attention was needed; and whether the bitches were reported to show any unusual behaviours (inappetence, discomfort, uncharacteristic irritability and uncharacteristic licking or chewing of other areas of the body). Postoperative complications were not defined further, and details were limited to text available in clinical records for LAP‐OVE bitches and to data provided in free‐text boxes for OVH‐CEL bitches. Data for wound healing were combined so that each bitch had a wound healing score out of 2, representing the total number of wound healing complications reported for the bitch. Similarly, data relating to unusual behaviours were combined so that each bitch had an “Unusual Behaviour” score out of 4.

Outcome variables examined were wound redness or swelling, wound discharge, wound healing score, inappetence, discomfort, irritability, licking or chewing of areas of the body, unusual behaviour score and complications requiring veterinary attention.

### Bias and study size

The statistical models for surgery duration included confounding factors.

Surgery duration for ovariohysterectomy in bitches has been reported to be impacted by bitch age and bodyweight (Burrow et al., 2005; Howe, 1997); therefore, these variables were included as covariates. All laparoscopic surgeries were undertaken by VP1. To investigate any difference in surgery duration related to multiple veterinary practices performing OVH‐CEL surgeries, data were analysed for all bitches and then for bitches neutered at VP1 only (LAP‐OVE = 213, OVH‐CEL = 121).

Study size was limited by the number of bitches for which data were available. Data availability was limited by the information reported on the data forms or in the practice records. The mean, SEM and confidence intervals (CI) were reported where appropriate.

### Quantitative variables and statistical methods

For each outcome variable, the number of bitches with data was described. Categorical covariates were examined, and any that had less than five bitches were grouped for analysis. Data were checked manually for obvious recording errors (one LAP‐OVE bitch with a calculated age at neutering of 0.5 months and a bodyweight of 30 kg was excluded from analyses involving age at neutering as a covariate). Generalised linear mixed models (GLMMs) were conducted using SPSS version 29 (IBM Corp., Armonk, NY, USA). Chi‐squared tests were run in XLStat version 2021 3.1 (Addinsoft, New York City, NY, USA). Results were considered significant when P < 0.05.

#### Perioperative outcomes

Bitch age and bodyweight at time of surgery were described for both surgery groups and were compared using Mann–Whitney tests of difference. Following preliminary analyses (all confounding variables were found to be significantly associated with surgery duration, P < 0.05), surgery duration (minutes) was examined as the continuous outcome variable using two GLMMs for a gamma distribution due to the skewed distribution of data: (1) including data from all four VP and (2) including data from VP1 only. Plots of residuals against predicted values were used to assess model fit. Surgery group (LAP‐OVE, OVH‐CEL), breed and complications during surgery were included as fixed factors. Multicollinearity was not detected between bodyweight and age (variance inflation factor = 1.140), and these variables were included as covariates. Veterinary practice (model for all VP) and veterinary surgeon were included as random effects.

The number of bitches in each group with intraoperative complications, complications during anaesthesia, during recovery from anaesthesia and in the immediate postoperative period were compared using chi‐squared tests when the numbers were sufficient for analysis. Types of analgesia at the time of surgery and any other drugs given were described as well as any medication patients were discharged with.

#### Postoperative period

Wound healing complications, complications that required veterinary attention and unusual behaviours that were reported either in the VP clinical records (for LAP‐OVE) or on the data capture forms (OVH‐CEL) were compared between groups using Chi‐squared tests. Surgery durations for bitches with and without wound healing complications were described.

## RESULTS

### Participants

Age at neutering was different between surgery groups (Mann–Whitney *U* = 45,329.5, P < 0.001). LAP‐OVE bitches were aged between 6.0 and 15.4 months (mean = 14.0 ± 7.8 months), and OVH‐CEL bitches were aged between 6.0 and 17.4 months (mean = 9.4 ± 3.4 months). One LAP‐OVE bitch had an incorrect age of 0.5 months recorded and therefore was excluded from the descriptive analysis for age and from any analyses involving age as a covariate. One VP (VP1) performed both LAP‐OVE (*n* = 213) and OVH‐CEL (*n* = 121) surgeries; three VPs performed only OVH‐CEL surgeries (VP2 *n* = 51, VP3 *n* = 66, VP4 *n* = 68).

Bodyweight data were recorded for 508 bitches (208 LAP‐OVE, 300 OVH‐CEL) at the time of surgery, and there was no difference in bodyweight between surgery groups (Mann–Whitney *U* = 30,813.0, P = 0.812). LAP‐OVE bitches weighed between 10.0 and 49.0 kg (mean 24.4 ± 0.5 kg); OVH‐CEL bitches weighed between 15.3 and 33.5 kg (mean 24.3 ± 0.2 kg).

Data for the perioperative period were available for 486 bitches (198 LAP‐OVE, 288 OVH‐CEL). Fifteen LAP‐OVE bitches had no data for surgery duration. Five OVH‐CEL bitches had no perioperative data; a further nine had data for perioperative complications but no surgery length data, and four only had data for surgery length with no data for perioperative complications.

Data for the postoperative period were available for 477 bitches (209 LAP‐OVE, 268 OVH‐CEL). Forty‐two bitches (4 LAP‐OVE, 38 OVH‐CEL) had no data for postoperative complications related to wound healing or unusual behaviour. Data for complications in the postoperative period that required veterinary attention were available for 515 bitches; four LAP‐OVE bitches had no data available.

### Perioperative outcomes

#### Surgery duration

Surgery duration was reported for 490 bitches (198 LAP‐OVE, 292 OVH‐CEL). LAP‐OVE surgeries ranged from 10 to 110 minutes (mean = 25.5 ± 0.8 minutes). OVH‐CEL surgeries ranged from 7 to 55 minutes (mean = 18.2 ± 0.4 minutes). Data for 13 of the 490 bitches (6 LAP‐OVE, 7 OVH‐CEL) were not included in the generalised linear models due to missing covariate information relating to bodyweight, intraoperative complications and having an incorrect age at the time of surgery.

When data for all VP (192 LAP‐OVE, 285 OVH‐CEL) were analysed using a GLMM, the overall model was significant (*F* = 29.640, DF = 9, P < 0.001). There was no difference in surgery duration between surgery groups (LAP‐OVE mean = 25.6 ± 0.8 minutes, OVH‐CEL mean = 18.1 ± 0.4 minutes; *F* = 3.551, DF = 1, P = 0.060). All other confounding variables included in the model were associated with surgery length (Table 2).

**Table 2:** Coefficients from the generalised linear mixed model for all surgeries assessing the effect of surgery group on surgery duration along with effects of fixed effects and covariates included in the model in comparison to the reference category (*)

Variable	Coefficient	SE	95% CI lower	95% CI upper	P
(Intercept)	3.016	0.129	2.762	3.269	0.000
Poodle cross	0.108	0.090	−0.068	0.285	0.229
Other	0.176	0.068	0.043	0.308	0.009
Labrador	0.030	0.067	−0.102	0.163	0.651
German shepherd dog and German shepherd dog cross	0.216	0.088	0.044	0.388	0.014
Golden retriever cross labrador	0.056	0.197	−0.331	0.444	0.775
Golden retriever*	–	–	–	–	–
OVH‐CEL	−0.359	0.191	−0.734	0.015	0.060
LAP‐OVE*	–	–	–	–	–
Intraoperative complications	0.520	0.077	0.369	0.671	<0.001
No intraoperative complications*	–	–	–	–	–
Age	0.081	0.030	0.022	0.139	0.007
Bodyweight	0.006	0.003	0.001	0.012	0.021

CI Confidence interval, LAP‐OVE Laparoscopic ovariectomy, OVH‐CEL Ovariohysterectomy via celiotomy

When data for VP1 surgeries only (192 LAP‐OVE, 109 OVH‐CEL) were analysed using a GLMM, the overall model was significant (*F* = 20.684, DF = 9, P < 0.001). There was no difference in surgery duration between surgery groups (LAP‐OVE mean = 25.6 ± 0.8 minutes, OVH‐CEL mean = 17.1 ± 0.6 minutes; *F* = 2.752, DF = 1, P = 0.098) and no effect of bodyweight. All other confounding variables included in the model were associated with surgery length (Table 3).

**Table 3:** Coefficients from the generalised linear mixed model for Veterinary Practice 1 (VP1) surgeries assessing the effect of surgery group on surgery duration, along with effects of fixed effects and covariates included in the model in comparison to the reference category (*)

Variable	Coefficient	SE	95% CI lower	95% CI upper	P
(Intercept)	3.064	0.145	2.780	3.348	0.000
Poodle cross	0.109	0.103	−0.095	0.312	0.295
Other	0.178	0.077	0.027	0.330	0.021
Labrador	0.028	0.077	−0.123	0.179	0.717
German shepherd dog and German shepherd dog cross	0.222	0.101	0.024	0.420	0.028
Golden retriever cross labrador	0.056	0.225	−0.387	0.499	0.803
Golden retriever*	–	–	–	–	–
OVH‐CEL	−0.361	0.218	−0.789	0.067	0.098
LAP‐OVE*	–	–	–	–	–
Intraoperative complications	0.635	0.128	0.383	0.887	<0.001
No intraoperative complications*	–	–	–	–	–
Age	0.079	0.035	0.009	0.148	0.027
Bodyweight	0.005	0.004	−0.002	0.012	0.125

CI Confidence interval, LAP‐OVE Laparoscopic ovariectomy, OVH‐CEL Ovariohysterectomy via celiotomy

#### Perioperative complications

Data on perioperative complications were available for 510 bitches for all surgeries (213 LAP‐OVE, 297 OVH‐CEL) and for 329 bitches for VP1 surgeries (213 LAP‐OVE, 116 OVH‐CEL). Intraoperative complications were reported for 14 bitches (5 [2.3%] LAP‐OVE, 9 [2.9%] OVH‐CEL; *χ* ^2^ = 0.217, DF = 1, P = 0.641) for all surgeries and for seven bitches (5 [2.3%] LAP‐OVE, 2 [1.7%] OVH‐CEL; Yates’ *χ* ^2^ = 0.001, DF = 1, P = 0.980) for VP1 surgeries. These were reported as: cervical stump double ligated (1 OVH‐CEL), religating both ovarian stumps with PDS (1 OVH‐CEL), right ovarian pedicle ligated twice (1 OVH‐CEL; due to high quantity of abdominal fat, 2 OVH‐CEL; due to bleeding), religating left ovarian pedicle due to bleeding (1 OVH‐CEL), larger incision made due to difficulty finding ovaries (1 OVH‐CEL), accidental digital perforation of spleen (1 OVH‐CEL), ovary disintegrated (1 LAP‐OVE), bleeding from right ovarian pedicle (1 LAP‐OVE) and bleeding (no further information on site of bleeding, 3 LAP‐OVE). One LAP‐OVE bitch was noted as “D in procedure” with no further detail to explain the complication.

Problems with anaesthesia were reported for two bitches (0 LAP‐OVE, 2 [0.8%] OVH‐CEL) for all surgeries and no bitches for VP1 surgeries. These were as follows: “became ‘light’ on clamping of the ovaries but settled well with briefly increased Isoflurane concentrations,” and requiring intravenous atropine due to a decrease in heart rate. Both responded well and had no other complications. Problems during recovery from anaesthesia were reported for three bitches (0 LAP‐OVE, 3 [1.0%] OVH‐CEL) for all surgeries and for two bitches (0 LAP‐OVE, 2 [1.7%] OVH‐CEL) for VP1 surgeries. These were reported as: becoming excitable (1 OVH‐CEL), vomiting (1 OVH‐CEL) and hypersalivation (1 OVH‐CEL). Statistical analysis was not possible due to low numbers of bitches reported with complications related to anaesthesia.

Postoperative complications prior to being discharged were reported for four bitches (1 [0.5%] LAP‐OVE, 3 [1.0%] OVH‐CEL; Yates’ *χ* ^2^ = 0.030, DF = 1, P = 0.862) for all surgeries and for one bitch (1 [0.5%] LAP‐OVE, 0 [0%] OVH‐CEL) for VP1 surgeries. These were reported as: spotting a small amount of blood from the vulva requiring overnight observations (1 OVH‐CEL) and a nose snuffle – acid reflux under GA (1 LAP‐OVE). Further detail was not provided for two OVH‐CEL bitches. Statistical analysis for VP1 surgeries was not possible due to the low number of bitches with complications.

No perioperative complications were reported for 97.2% of LAP‐OVE, 94.3% of all OVH‐CEL bitches and 96.6% for VP1 OVH‐CEL. No bitch had more than one perioperative complication; therefore, no bitch had a perioperative complication score greater than “1.” Perioperative complication scores were “0” for 207 LAP‐OVE and 280 OVH‐CEL bitches and were “1” for six LAP‐OVE (2.8%) and 17 OVH‐CEL bitches (5.7%) (*χ* ^2^ = 2.434, DF = 1, P = 0.119).

#### Analgesia and other medications

Five hundred and eight bitches had a type of analgesia reported (209 LAP‐OVE, 299 OVH‐CEL). This was most frequently a combination of NSAIDs and opioids (LAP‐OVE 100%, OVH‐CEL 98.3%) using buprenorphine (used in 433 surgeries), butorphanol (used in two surgeries), carprofen (used in 66 surgeries), meloxicam (used in 232 surgeries) or methadone (used in 69 surgeries). All bitches that had a perioperative complication reported (6 LAP‐OVE, 17 OVH‐CEL) received a combination of opioid and NSAID analgesia. The two bitches reported with problems with anaesthesia, and two of the three bitches reported with problems during recovery from anaesthesia had either carprofen or meloxicam with buprenorphine. The third bitch with problems during recovery from anaesthesia (hypersalivating) had meloxicam and methadone.

### Postoperative outcomes

#### Wound healing

Data for redness or swelling of the wound were reported for 477 bitches (209 LAP‐OVE, 268 OVH‐CEL) for all surgeries and for 318 bitches for VP1 surgeries (209 LAP‐OVE, 109 OVH‐CEL). There were fewer LAP‐OVE than OVH‐CEL bitches with redness or swelling of the wound site for all surgeries (34 [16.3%] LAP‐OVE, 98 [36.6%] OVH‐CEL; *χ* ^2^ = 24.175, DF = 1, P < 0.001), and for VP1 surgeries (34 [16.3%] LAP‐OVE, 47 [43.1%] OVH‐CEL; *χ* ^2^ = 27.208, DF = 1, P < 0.001). Surgery durations for bitches with redness or swelling of the wound were between 8 and 60 minutes (mean = 19.6 ± 0.7 minutes) compared to between 7 and 60 minutes (mean = 21.6 ± 0.5 minutes) for bitches without redness or swelling of the wound reported.

Data for wound discharge were reported for 471 bitches (209 LAP‐OVE, 262 OVH‐CEL) for all surgeries and for 314 bitches for VP1 surgeries (209 LAP‐OVE, 105 OVH‐CEL). For all surgeries, there were seven [3.3%] LAP‐OVE bitches and 16 [6.1%] OVH‐CEL bitches with wound discharge reported (*χ* ^2^ = 1.903, DF = 1, P = 0.168). For VP1 surgeries, there were seven [3.3%] LAP‐OVE and five [4.8%] OVH‐CEL bitches (Yates’ *χ* ^2^ = 0.092, DF = 1, P = 0.762). Surgery duration for bitches with a wound discharge was between 8 and 35 minutes (mean = 21.7 ± 1.8 minutes) compared to between 7 and 60 minutes (mean = 21.0 ± 0.4 minutes) for bitches without a wound discharge reported.

A wound healing score for combined data relating to wound redness or swelling and wound discharge was awarded to 475 bitches for all surgeries (209 LAP‐OVE, 266 OVH‐CEL) and to 316 bitches for VP1 surgeries (209 LAP‐OVE, 107 OVH‐CEL). For all surgeries (*χ* ^2^ = 22.476, DF = 1, P < 0.001) and VP1 surgeries (*χ* ^2^ = 24.347, DF = 1, P < 0.001), more LAP‐OVE than OVH‐CEL bitches scored “0” and less scored “1” or more (Table 4).

**Table 4:** The number (and percentage) of bitches neutered via laparoscopic ovariectomy (LAP‐OVE) or ovariohysterectomy via celiotomy (OVH‐CEL) with each wound healing score for the postoperative period

Wound healing score	LAP‐OVE	All OVH‐CEL surgeries	VP1 OVH‐CEL surgeries
0	170 (81.3%)	163 (61.3%)	59 (55.1%)
1	37 (17.7%)	92 (34.6%)	44 (41.1%)
2	2 (1.0%)	11 (4.1%)	4 (3.7%)

#### Complications requiring veterinary attention

For all surgeries, data were available for 209 LAP‐OVE and 306 OVH‐CEL bitches relating to complications requiring veterinary attention: 27 LAP‐OVE (12.9%) and 50 OVH‐CEL (16.3%) bitches required veterinary attention (*χ* ^2^ = 1.143, DF = 1, P = 0.285; Table 5). For VP1 surgeries, data were available for 209 LAP‐OVE and 121 OVH‐CEL bitches, and fewer LAP‐OVE bitches (27 [12.9%]) than OVH‐CEL (29 [24.0%]) bitches required veterinary attention (*χ* ^2^ = 6.639, DF = 1, P = 0.010; Table 6). Complications requiring veterinary attention for all surgeries and for VP1 surgeries were most commonly related to the wound, including swelling, discharge, infection, suture reaction or abscess (for all surgeries: *n* = 39; 6 LAP‐OVE, 33 OVH‐CEL, for VP1: *n* = 7, 5 Lap‐OVE, 2 OVH‐CEL). For all surgeries, six LAP‐OVE and 27 OVH‐CEL bitches developed a seroma. Vomiting, loose stool or diarrhoea were reported in 15 bitches (12 LAP‐OVE, 3 OVH‐CEL).

**Table 5:** The number (and percentage) of bitches neutered via laparoscopic ovariectomy (LAP‐OVE) or ovariohysterectomy via celiotomy (OVH‐CEL) with each complication or unusual behaviour reported in the postoperative period

Complication or behaviour reported	Number of bitches with data available (LAP‐OVE, OVH‐CEL)	Total number affected (%)	LAP‐OVE affected (%)	OVH‐CEL affected (%)	*χ* ^2^	P
Wound redness or swelling	209, 268	132 (27.7%)	34 (16.3%)	98 (36.6%)	24.175	<0.001
Wound discharge	209, 262	23 (4.9%)	7 (3.3%)	16 (6.1%)	1.903	0.168
Complications that required veterinary attention	209, 306	77 (15.0%)	27 (12.9%)	50 (16.3%)	1.143	0.285
Inappetence	209, 256	31 (6.7%)	5 (2.4%)	26 (10.2%)	11.147	<0.001
Discomfort after surgery	209, 253	61 (13.2%)	16 (7.7%)	45 (17.8%)	10.250	<0.001
Uncharacteristic signs of irritability	209, 255	7 (1.5%)	1 (0.5%)	6 (2.4%)	1.601	0.206
Uncharacteristic licking or chewing	209, 254	28 (6.0%)	6 (2.9%)	22 (8.7%)	6.766	<0.001

Results of Chi‐square analysis are presented (*χ* ^2^, P)

**Table 6:** The number (and percentage) of bitches neutered via laparoscopic ovariectomy (LAP‐OVE) or ovariohysterectomy via celiotomy (OVH‐CEL) by VP1 with each complication or unusual behaviour reported in the postoperative period

Complication or behaviour reported	Number of bitches with data available (LAP‐OVE, OVH‐CEL)	Total number affected (%)	LAP‐OVE affected (%)	OVH‐CEL affected (%)	*χ* ^2^	P
Wound redness or swelling	209, 109	81 (25.5%)	34 (16.3%)	47 (43.1%)	27.208	<0.001
Wound discharge	209, 105	12 (3.8%)	7 (3.3%)	5 (4.8%)	0.092	0.762
Complications that required veterinary attention	209, 107	56 (17.0%)	27 (12.9%)	29 (24.0%)	6.639	0.010
Inappetence	209, 105	19 (6.1%)	5 (2.4%)	14 (13.3%)	14.716	<0.001
Discomfort after surgery	209, 102	30 (9.7%)	16 (7.7%)	14 (13.7%)	2.898	0.089
Uncharacteristic signs of irritability	209, 103	4 (1.3%)	1 (0.5%)	3 (2.9%)	1.593	0.207
Uncharacteristic licking or chewing	209, 103	16 (5.1%)	6 (2.9%)	10 (9.7%)	6.631	0.010

Results of Chi‐square analysis are presented (*χ* ^2^, P)

#### Unusual behaviour

The number of bitches with data for each unusual behaviour (inappetence, discomfort, irritability and licking or chewing of areas of the body) reported in the postoperative period was 209 for LAP‐OVE bitches, between 253 and 256 for OVH‐CEL bitches following all surgeries, and between 102 and 105 for OVH‐CEL bitches following VP1 surgeries (Tables 5 and 6).

For all surgeries (Table 5) and for VP1 surgeries (Table 6), each individual unusual behaviour was noted for numerically fewer LAP‐OVE than OVH‐CEL bitches. Differences were significant between LAP‐OVE and all OVH‐CEL bitches for inappetence (*χ* ^2^ = 11.147, DF = 1, P < 0.001), discomfort (*χ* ^2^ = 10.250, DF = 1, P < 0.001) and for uncharacteristic licking or chewing of other areas of the body (*χ* ^2^ = 6.766, DF = 1, P < 0.001). Comparisons for VP1 surgeries identified significant differences between LAP‐OVE and OVH‐CEL bitches for inappetence (*χ* ^2^ = 14.716, DF = 1, P < 0.001) and for uncharacteristic licking or chewing of other areas of the body (*χ* ^2^ = 6.631, DF = 1, P = 0.010).

When data were combined into an unusual behaviour score for all surgeries, 191 LAP‐OVE (91.4%) and 172 OVH‐CEL bitches (67.5%) had a score of “0,” 17 LAP‐OVE (8.1%) and 69 OVH‐CEL bitches (27.0%) had a score of “1,” and one LAP‐OVE (0.5%) and 14 OVH‐CEL bitches (5.5%) had a score of “2” or more. For VP1 surgeries, 191 LAP‐OVE (91.4%) and 68 OVH‐CEL bitches (66.0%) had a score of “0,” 17 LAP‐OVE (8.1%) and 30 OVH‐CEL bitches (29.1%) had a score of “1,” and one LAP‐OVE (0.5%) and five OVH‐CEL bitches (4.9%) had a score of “2” or more. For all surgeries (*χ* ^2^ = 38.646, DF = 1, P < 0.001) and VP1 surgeries (*χ* ^2^ = 31.488, DF = 1, P < 0.001), more LAP‐OVE than OVH‐CEL bitches scored “0” and less scored “1” or more. Two OVH‐CEL bitches had three unusual behaviours reported: both showed inappetence and uncharacteristic irritability; one also showed licking or chewing of the leg/flank, and one showed discomfort. Of the other 27 bitches that showed licking or chewing parts of their body and the location on the body was reported, LAP‐OVE bitches only showed licking of the wound (*n* = 3). In OVH‐CEL bitches, licking and chewing included the vulva, leg, tail, feet, flank (often in combination with leg) and abdomen.

## DISCUSSION

This study examined surgery length and the peri‐ and postoperative outcomes in bitches neutered via laparoscopic ovariectomy or open ovariohysterectomy (celiotomy). These are anecdotally reported as the two most commonly performed neutering procedures for bitches in the UK. Bitches neutered by laparoscopic ovariectomy or ovariohysterectomy by celiotomy had similar surgery durations and a low incidence of perioperative complications. Fewer bitches neutered by laparoscopic ovariectomy had wound healing complications, complications requiring veterinary attention and unusual behaviours reported in the postoperative period than those neutered by open ovariohysterectomy.

The study was limited by the methods of data collection. Purpose‐designed data collection forms for peri‐ and postoperative outcomes were used for bitches neutered by ovariohysterectomy, whereas data for bitches neutered by laparoscopic ovariectomy were collected directly from the veterinary practice records. This could have led to underreporting of some complications in the peri‐ and postoperative periods for laparoscopic surgeries, and further study using the same data collection forms for both groups would be required to confirm the results presented here. However, complications during surgery and those in the postoperative period that required veterinary intervention were less likely to be affected by the methods of data collection. The perioperative complications that required reporting were not defined; therefore, the results may be affected by differences between veterinarians in what was considered a complication. The study design aimed to include bitches of comparable breed and body size, and bodyweight was not different between the two surgical groups. However, age at surgery was different. While this confounder was controlled for in the analyses for surgery duration, it was not possible to control for this variable in the analysis of data for other peri‐ and postoperative outcomes due to small numbers of bitches reported with the outcomes. Age at surgery has been associated with the occurrence of complications by other authors (Howe, 1997; Pollari et al., 1996; Pollari & Bonnett, 2006); therefore, there may be a confounding effect of age on these variables that would need to be investigated in future studies. Additionally, all laparoscopic surgeries were performed at VP1 by experienced surgeons, which could have led to a bias in results for bitches neutered laparoscopically. However, data were analysed and presented including surgeries by all four practices and by VP1 separately so that any differences could be highlighted. The results presented here for VP1 offer a valid comparison of surgery durations for the two surgical methods that are not influenced by unknown or inconsistent surgeon experience.

Significant differences in surgery length between ovariohysterectomy and ovariectomy surgeries, and between laparoscopic and open surgeries, have been reported for bitches in previous studies. Ovariohysterectomies, whether performed laparoscopically or by celiotomy, are reported with longer surgical times than ovariectomies (Corriveau et al., 2017; Tallant et al., 2016). Longer laparoscopic than open surgery durations have been reported for both ovariectomy and ovariohysterectomy surgeries by some authors (Culp et al., 2009; Davidson et al., 2004) but not by others (Shariati et al., 2014). However, these studies did not consider confounding variables in the analyses. Others, that accounted for confounders, suggested no difference in surgery length (Devitt et al., 2005; Peeters & Kirpensteijn, 2011). In the present study, although reported laparoscopic surgery durations were longer (mean of 25 minutes) than surgeries performed by celiotomy (mean of 17 to 18 minutes), these differences were not significant in models that included covariates. However, all other variables included in the model for all surgeries, and all other variables except for bodyweight in the model for VP1 surgeries, were associated with surgery duration. This highlights the importance of including these potential confounders in statistical analysis for surgery duration and the potential limitations of the findings from other studies that failed to do so.

Longer surgery durations for surgeries with intraoperative complications and with increasing age of the bitch, as reported in the present study, were anticipated based on the findings of others (Howe, 1997; Muraro & White, 2014). The differences in surgery duration according to bitch breed, in models that controlled for the effects of other potential confounders, may be less expected. In the present study, in both models, German shepherd dogs and their crosses and “other” breeds had significantly longer surgery durations than the reference breed, the golden retriever (see Tables 2 and 3). Further work is required to elucidate the reasons for this.

Within the present study, perioperative complication rates were low (less than 3% for any complication) and were not different between the two groups. Most bitches (97.2% LAP‐OVE, 91.5% OVH‐CEL) had no perioperative complications reported. This is in accordance with other studies that have reported rates between 1% and 20%, most commonly lower than 10% (Airikkala‐Otter et al., 2018; Belluzzi et al., 2022; Burrow et al., 2005; Corriveau et al., 2017; Muraro & White, 2014). The proportion of bitches with intraoperative complications for LAP‐OVE (2.3%) was comparable to those reported for experienced surgeons by others (1.3% to 1.7%; Pope & Knowles, 2014). The choice of opioid may have provided different levels of analgesia (Warne et al., 2013) and therefore may have impacted perioperative complications. Only one bitch became light during the procedure, and one became excitable during recovery from anaesthesia; both bitches received Meloxicam and Buprenorphine. In the immediate postoperative period, none of the complications would have been related to the analgesia used.

Postoperative complications were more commonly reported than perioperative complications, with wound healing the most frequently reported problem, similar to the work of others (Adin, 2011; Burrow et al., 2005; Muraro & White, 2014). Within the LAP‐OVE and OVH‐CEL groups, there were two methods of skin closure used: permanent non‐absorbable or synthetic absorbable sutures. It is possible that wound healing may have been affected by the differences in suture methods. However, a lack of recording for individual surgeries meant that this could not be examined and would be useful to consider in future studies. Fewer LAP‐OVE than OVH‐CEL bitches were reported to have postoperative complications in the days following surgery. Considering wound healing specifically, there were over twice as many bitches with wound redness or swelling reported following OVH‐CEL for all surgeries (36.3%) and for VP1 surgeries (43.1%) compared to the LAP‐OVE surgery group (16.3%). Similar findings were reported by Charlesworth and Sanchez (2019) in a descriptive study comparing complications post‐surgery between bitches following laparoscopic versus open ovariectomy. In that study, overall and wound healing complications were reported following 20% and 11% of laparoscopic and 44% and 28.3% of open surgeries, respectively. Other authors have reported no difference in wound complications between bitches neutered by laparoscopic ovariectomy versus laparoscopic ovariohysterectomy (Corriveau et al., 2017).

While the occurrence of unusual behaviours in the days following neutering surgeries performed using different methods in the bitch has been less well‐studied than physical outcomes post‐surgery, some studies have shown differences in behavioural responses. Pain in the days following surgery can lead to behavioural changes being observed (Väisänen et al., 2004). In two studies, pain scores were lower for bitches undergoing laparoscopic compared with open ovariohysterectomy surgeries (Davidson et al., 2004; Devitt et al., 2005). Differences in the change of total activity counts after surgery (25% decrease in total activity counts following laparoscopic ovariectomy versus 62% decrease in total activity counts following open ovariectomy) have been reported (Culp et al., 2009), suggesting changes in behaviour post‐surgery that are greater for open neutering surgeries. In the present study, four unusual behaviours were examined. For all surgeries and VP1 surgeries, there were more OVH‐CEL bitches with inappetence and uncharacteristic licking or chewing other areas of the body reported than LAP‐OVE bitches. Additionally, for all surgeries, there were more OVH‐CEL than LAP‐OVE bitches with discomfort (Tables 5 and 6). While these data suggest that higher proportions of the behaviours were observed in bitches following ovariohysterectomy by celiotomy than in bitches neutered by laparoscopic ovariectomy, it is possible that the result is exaggerated by the inconsistent data collection methods. These behaviours may be the variable most likely to be underreported in veterinary practice records compared to on forms that ask specific questions relating to the behaviours.

Further investigation into the peri‐ and postoperative complications observed in bitches neutered using these two commonly used surgical methods using the detailed data capture forms that have been previously described (Moxon et al., 2023) would be useful to confirm the results presented here. However, this study suggests that when experienced surgeons perform laparoscopic ovariectomy, peri‐ and postoperative outcomes are favourable in comparison with open ovariohysterectomy (celiotomy).

This is the first study to examine surgery duration and surgical outcomes for the two most commonly performed neutering surgeries for bitches: laparoscopic ovariectomy and open ovariohysterectomy (celiotomy). When potential confounders were included in an analysis for surgery duration, there was no difference in length of surgery, defined as “skin to skin,” between the two surgical methods. The findings provide further evidence to support the contention that postoperative complications, including those related to wound healing and those requiring further veterinary intervention, are lower for laparoscopic than open neutering surgeries in bitches. These findings are of interest to veterinarians and pet dog owners, in terms of improving the understanding of impacts of different surgical methods on postoperative recovery in order to make informed choices when neutering bitches.

## Author contributions

**R. Moxon:** Conceptualisation; Data curation; Formal analysis; Investigation; Methodology; Project administration; Resources; Validation; Visualisation; Writing – original draft; Writing – review & editing. **E. Yarwood**: Data curation; Formal analysis; Investigation; Methodology; Writing – original draft. **G. C. W. England:** Conceptualisation; Methodology; Validation; Supervision; Writing – review & editing. **J. Came**: Data curation; Writing – original draft; Writing – review & editing. **H. Hawkins:** Data curation.

## Conflict of interest

No conflicts of interest have been declared.

## Data Availability

The data that support the findings of this study are available on request from the corresponding author. The data are not publicly available due to privacy or ethical restrictions.
